# Does the rising placebo response impact antihypertensive clinical trial outcomes? An analysis of data from the Food and Drug Administration 1990-2016

**DOI:** 10.1371/journal.pone.0193043

**Published:** 2018-02-28

**Authors:** Arif Khan, Kaysee Fahl Mar, Joshua Schilling, Walter A. Brown

**Affiliations:** 1 Northwest Clinical Research Center, Bellevue, Washington, United States of America; 2 Department of Psychiatry, Duke University School of Medicine, Durham, North Carolina, United States of America; 3 Department of Cardiology, Baystate Medical Center, University of Massachusetts, Springfield, Massachusetts, United States of America; 4 Department of Psychiatry and Human Behavior, Brown University, Providence, Rhode Island, United States of America; Royal College of Surgeons in Ireland, IRELAND

## Abstract

**Background:**

Recent studies show that placebo response has grown significantly over time in clinical trials for antidepressants, ADHD medications, antiepileptics, and antidiabetics. Contrary to expectations, trial outcome measures and success rates have not been impacted. This study aimed to see if this trend of increasing placebo response and stable efficacy outcome measures is unique to the conditions previously studied or if it occurs in trials for conditions with physiologically-measured symptoms, such as hypertension.

**Method:**

For this reason, we evaluated the efficacy data reported in the US Food and Drug Administration Medical and Statistical reviews for 23 antihypertensive programs (32,022 patients, 63 trials, 142 treatment arms). Placebo and medication response, effect sizes, and drug-placebo differences were calculated for each treatment arm and examined over time using meta-regression. We also explored the relationship of sample size, trial duration, baseline blood pressure, and number of treatment arms to placebo/drug response and efficacy outcome measures.

**Results:**

Like trials of other conditions, placebo response has risen significantly over time (*R*^2^ = 0.093, p = 0.018) and effect size (*R*^2^ = 0.013, p = 0.187) drug-placebo difference (*R*^2^ = 0.013, p = 0.182) and success rate (134/142, 94.4%) have remained unaffected, likely due to a significant compensatory increase in antihypertensive response (*R*^2^ = 0.086, p<0.001). Treatment arms are likely overpowered with sample sizes increasing over time (*R*^2^ = 0.387, p<0.0001) and stable, large effect sizes (0.78 ±0.37). The exploratory analysis of sample size, trial duration, baseline blood pressure, and number of treatment arms yielded mixed results unlikely to explain the pattern of placebo response and efficacy outcomes over time. The magnitude of placebo response had no relationship to effect size (p = 0.877), antihypertensive-placebo differences (p = 0.752), or p-values (p = 0.963) but was correlated with antihypertensive response (*R*^2^ = 0.347, p<0.0001).

**Conclusions:**

As hypothesized, this study shows that placebo response is increasing in clinical trials for hypertension without any evidence of this increase impacting trial outcomes. Attempting to control placebo response in clinical trials for hypertension may not be necessary for successful efficacy outcomes. In exploratory analysis, we noted that despite finding significant relationships, none of the trial or patient characteristics we examined offered a clear explanation of the rise in placebo and stability in outcome measures over time. Collectively, these data suggest that the phenomenon of increasing placebo response and stable efficacy outcomes may be a general trend, occurring across trials for various psychiatric and medical conditions with physiological and non-physiological endpoints.

## Introduction

Although the placebo effect is a powerful tool for the treatment of patients with both psychiatric and physical illnesses, the placebo response as a measurement of these non-pharmacological effects in clinical trials has historically been viewed as a problem in the context of these trials [1]. Following the finding by Walsh et al in 2001 [2] that the placebo response in clinical trials for depression was variable and growing, an assumption emerged that such growth in placebo response was likely responsible for the low success rate and poor efficacy outcomes seen in antidepressant trials [3]. However, recent analysis has shown that this assumption is no longer tenable. While the placebo response is still rising significantly, negative impacts on the efficacy outcomes of antidepressant clinical trials have not been observed [4]. Effect size, drug-placebo differences, and success rate have remained stable, due to a parallel increase in drug response. This pattern of rising placebo response and unaffected trial outcomes does not appear to be unique to antidepressant trials; we have also seen it in clinical trials for ADHD medications [5], antiepileptics [6], and antihyperglycemics [7].

In this context, it is important to note that other investigators have questioned if the placebo response is actually rising in antidepressant trials [8]. Specifically, these authors used a categorical definition of placebo response (number of responders, those with 50% reduction in symptoms from baseline). However, this is a transformed endpoint which is not used by regulatory agencies like the US FDA in their assessment of pharmacological treatments. This categorical assay of placebo response, along with the fact that these authors grouped the trials by five-year intervals, drastically reduces the sensitivity of their analysis. Considering this and other significant divergence in methodological decisions in these investigators’ analysis, we conclude from our previous analysis [4] that the magnitude of placebo response as measured continuously over time in FDA reviewed clinical trials of antidepressants, has definitely increased.

Given the aforementioned findings, we decided to evaluate if this pattern of rising placebo response and stable efficacy outcomes over time is exclusive to clinical trials of psychiatric conditions like depression or ADHD or medical conditions like epilepsy or diabetes, or if this pattern could be seen in other conditions like hypertension. To evaluate this possibility, we examined efficacy data from the New Drug Approval packets for investigational antihypertensives. We chose hypertension trials because they are prone to a non-trivial placebo response [9–11]. Additionally, hypertension trial designs are fairly consistent and they evaluate efficacy over a period of weeks. And most importantly, a systematic analysis of primary-sourced FDA clinical trial efficacy data for hypertension trials has not be undertaken as of yet, representing a considerable gap in the literature.

While hypertension trials have many design similarities that make them comparable to trials we have analyzed for the aforementioned conditions, it is important to consider that hypertension trials have some notable idiosyncrasies. One such idiosyncrasy we considered stems from the fact that the selected primary efficacy outcome measure is thought to potentially influence hypertension trial outcomes. This is based on the idea that placebo response may vary across different contexts and styles of blood pressure measurement. Studies [12] have suggested that the “white-coat effect” on in-office blood pressure measurement may contribute significantly to the placebo response. Changing the context and increasing data points by using more frequent out-of-office measurements, such as 24-hour ambulatory blood pressure cuffs or in-home self-monitoring, may reduce the statistical noise of normal blood pressure variability. Such techniques may therefore increase reproducibility [13–15] and yield lower estimations of placebo response [16–19]. The adoption of such techniques in the measurement of primary efficacy endpoints in FDA clinical trials has not been quantified.

Additionally, it is important to note that hypertension trials tend to have much larger sample sizes than antidepressant and ADHD trials. Smaller trials (less than 100 patients) are infrequent in the recent history of antihypertensive trials. Given that we have found in previous analysis that the adequacy of statistical power from sample sizes has had a significant effect on the relationship between placebo response and trial outcomes in antidepressant trials, we aimed to explore the impact of large trial sample sizes as it relates to hypertension trial outcomes.

To investigate the placebo response and trial efficacy outcomes for antihypertensives, we evaluated the clinical trial data submitted as proof of efficacy and reviewed by the US Food and Drug Administration for 23 antihypertensive medications between 1990 and 2016. Our hypothesis was that the magnitude of placebo response in clinical trials of antihypertensive medications has increased over time without impacting the effect size, drug-placebo difference, or the success rate of these trials. We presumed that this pattern would occur due to a compensatory increase in the magnitude of response in the antihypertensive treatment group over time. We also explored the relationships of trial duration, number of treatment arms, baseline blood pressure, and sample size to efficacy outcomes and placebo response to see if changes in these variables could adequately explain any changes that occurred over time.

## Method

### Source: FDA Access Data database

We used the New Drug Approval (NDA) packets published on the US FDA database (http://www.accessdata.fda.gov/) [20] as our source for efficacy data. A benefit of this database is that these data have been unbiasedly reviewed for approval by FDA medical and statistical staff as compared to data from published reports [21]. Additionally, the statistical treatments and presentation of data in these reviews are of sufficient quality, completeness, and comparability such that we could analyze these efficacy data across different types of investigational agents.

### Selection of programs

We selected programs for investigational antihypertensive medications (oral agents indicated for treatment of essential hypertension) if their NDAs (which include the FDA medical and statistical reviews of the trials conducted for efficacy evaluation) were available on the FDA database website (http://www.accessdata.fda.gov/).

Programs for which multiple indications were listed were only included if the trials submitted for proof of efficacy used patients diagnosed exclusively with essential hypertension. Combinations (ie. + HCT) and new formulations (ie. extended release formulations) were included if the cited trial data were not already included in a New Drug Approval packet for a previous formulation.

### Selection of trials/treatment arms

For inclusion in this current analysis, we considered all of the trials reviewed for efficacy from each NDA program that we could access. Of these trials, we included all acute, placebo-controlled trials of approved doses of the investigational oral antihypertensive that were cited in the integrated review of efficacy for approval and met the following PICO criteria: P: adults, aged 18–65 years, with essential hypertension (defined as diastolic blood pressure ≥90 mmHg), inclusive of both male and female patients of all races. I: oral antihypertensive drugs at approved dosing levels. C: placebo pill. O: either diastolic or systolic (whichever was indicated as the primary outcome measure), seated or supine blood pressure measured after a duration of ≥3 weeks and ≤24 weeks after baseline measurements. Studies with incomparable design differences (ie. relapse prevention studies) were also excluded. The patients in these FDA studies were otherwise healthy or had all other physical illnesses under adequate pharmaceutical control.

It is important to note that sub-therapeutic doses are intentionally included in dose-finding studies in order to demonstrate the lowest effective dose. Because these treatment arms serve a purpose other than to be approved at the dose used, we excluded such treatment arms using unapproved doses of the active medication.

### Data collection—Extraction from FDA efficacy review

FDA reviewers conduct independent statistical analysis of efficacy for each treatment arm at different dose levels within a trial. For this reason, we decided to examine treatment arms independently of the trials they were in. Within the NDA, efficacy endpoint analysis is conducted which compares symptom reduction between antihypertensive and placebo treated patients on the pre-specified primary outcome measure. The results from these analyses are typically presented in a table. We extracted in duplicate form the baseline and change scores for both drug and placebo, and the p-value resulting from the comparison of change scores between active treatment and control.

P-values: P-values were recorded in exact form from the endpoint analysis conducted by the FDA statistical reviewer. P-values were reported by the FDA for each individual trial arm comparison of antihypertensive treatment to placebo. In cases of both significant (p < 0.05) and insignificant (p > 0.05) p-values, we recorded the p-value along with all of the decimal places reported rather than the threshold (ie. p = 0.034 rather than p<0.05). In some cases, only the threshold was reported in the NDA and so we recorded the threshold.

Baseline Scores: Mean blood pressure at the beginning of the trial was reported for placebo and antihypertensive-treated patients. These baseline scores were extracted for each treatment arm.

Drug and Placebo Response: Drug/placebo response was defined as the change in the primary efficacy measure of blood pressure. Change scores for placebo and active treatment represented the reported mean reductions in blood pressure points between start and end scores at the conclusion of the treatment period (Baseline mean BP—Endpoint mean BP). Evaluation of such change scores is what FDA reviewers use to determine the efficacy of investigational agents. For the purposes of this analysis, change scores are expressed as a positive number if treatment reduced blood pressure and are negative if blood pressure increased. Higher change scores indicated greater treatment response.

Trial Arm Success: FDA reviewers use p-value < 0.05 to determine statistical success of a treatment arm comparison of drug to placebo. Treatment arms were denoted as failed if the resulting p-value was ≥ 0.05 for the comparison. The success rate was the number of treatment arms meeting statistical significance as reported by the FDA out of the total number of treatment arms in the trials.

Treatment Arm Sample Size: Sample size was calculated by adding the reported number of Intent-to-treat (ITT) patients from placebo treatment (placebo n) to the number of ITT patients from active treatment (antihypertensive n) to generate a single N value for the total sample in the treatment arm comparison. Each treatment arm had a Sample Size N comprised of placebo cell n and active treatment n.

### Efficacy outcome measures

Drug-Placebo Difference: The difference in treatment response between placebo and antihypertensives was calculated by subtracting the placebo change score from the active treatment change score for each treatment arm.

Effect Size: We used Hedges’ G formula to calculate a standardized effect size for the drug-placebo difference in blood pressure reduction (sometimes referred to as the placebo-subtracted treatment effect). In cases where sufficient variance estimations were reported (ie. standard deviation or standard error and sample size), we calculated effect size using the typical formula. In cases where no measures of variance were given, we used a workaround method of Corrected Hedges’ G formula which uses precise p-values, following in suit with Turner et al [20]:

#### Hedge’s G workaround method using precise P-values

As proposed by Turner et al [20] in their study of antidepressant clinical trial data, this method utilizes the Inverse T- score function (TINV) in Microsoft Excel. Precise p-values (most decimal places given) and degrees of freedom are imputed into the function to calculate a t-score, which can be transformed to Hedges’ G using the following equation: $g=\text{t}\;\text{x}\;\sqrt{\frac{1}{n_{\left(drug\right)}}+\frac{1}{n_{\left(placebo\right)}}}$. Hedges’ G effect size has a proposed correction for small sample size as follows: *Corrected $g=g\;\text{x}\;\left(1-\frac{3}{4\left(\text{df}\right)-1}\right)$*.

### Statistical measures

Statistical measures were generated with IBM Statistical Package for the Social Sciences (SPSS). Simple meta-regression analysis was used to predict treatment response and outcomes based on year of approval and to plot the data over time. Meta-regression with random effects via maximum likelihood was modeled to evaluate potential modifiers of placebo and drug response and efficacy outcome measures.

#### Trial and patient characteristics

We recorded the duration for each trial as the number of weeks between baseline measurement and the final measurement of blood pressure. We were also able to record the number of treatment arms included in each trial (as originally designed, including active comparators). For example, a trial with placebo, two different dose levels of investigational antihypertensive, and an active comparator arm would have been coded as having four treatment arms. Patient characteristics including proportion of male/females, racial demographics, and mean age could not be statistically analyzed due to the fact that less than two-thirds of the trials reported these measures. Duration, number of treatment arms, baseline blood pressure (severity of hypertension), year of approval, and sample size were able to be entered as potential modifiers in the meta-regression models.

## Results

There were 23 antihypertensive medications (year of approval) that met inclusion for this study: isradipine (1990), eprosartan mesylate (1997), valsartan (1997), telmisartan (1998), candesartan cilexetil (1998), valsartan HCT (1998), telmisartan HCT (2000), eprosartan HCT (2000), candesartan HCT (2001), olmesartan medoxomil (2002), eplerenone (2002), olmesartan HCT (2003), amlodipine besylate + olmesartan medoxomil (2007), nebivolol hydrochloride (2007), aliskiren hemifumarate (2007), aliskiren HCT (2007), aliskiren + valsartan (2009), amlodipine + and telmisartan (2009), aliskiren + amlodipine (2010), azilsartan kamedoxomil (2011), azilsartan (2011), perindopril + amlodipine (2015), and nebivolol + valsartan (2016).

We excluded 11 trials of subgroups including four trials evaluating males only, two trials of elderly patients (+65 yo), and five trials of only racial subgroups. We also excluded four trials with incomparable design differences (ie relapse prevention trials), eight trials with durations < 3 weeks or > 24 weeks, and four using alternative outcome measures (such as cough studies). We also excluded 26 trials that were not placebo-controlled. Exclusion of these trials left 63 trials for analysis.

From these trials, 211 treatment arms reported outcome data. After excluding 69 treatment arms with unapproved dose levels, 142 treatment arms remained for this analysis.

### Table 1: Summary of antihypertensive treatment arm data

**Table 1:** Summary of data from 63 clinical trials (142 treatment arms) conducted for efficacy approval by the US FDA for 23 antihypertensive medications between 1990 and 2016. Protocol Number, Trial Duration, Primary Efficacy Blood Pressure Measure, Mean Baseline and Change Scores on Primary Efficacy Measure, Number of Patients Per Treatment Cell, P-Values, and Effect Size^a^.

Protocol Number ^Duration (weeks)^	Primary Efficacy Measure (si = Sitting, su = Supine, Amb = ambulatory)	Placebo Baseline/Change Score [N Patients]	Investigational Antihypertensive Baseline/Change Score [N Patients]	Reported P-Value of Endpoint Analysis and Effect Size (Hedges’ G)
**Isradipine (1990)**				
7 ^4 wks^	SuDBP	100.5/1.5 [11]	**99.8/13.2 [12]**	p = 0.0051 (1.26)
11 ^3 wks^	SuDBP	103.4/3.8 [12]	**102.3/14.8 [12]**	p = 0.0007 (1.54)
301 ^5 wks^	SuDBP	103.9/6.1 [41]	**104.2/13.8 [40]**	p = 0.0001 (0.91)
			**104.8/15.8 [39]**	p = 0.0001 (1.11)
			**103.5/17.2 [41]**	p = 0.0001 (1.34)
			**103.5/17.1 [41]**	p = 0.0001 (1.32)
302 ^4 wks^	SuDBP	103.2/5.1 [49]	**103.5/12.9 [49]**	p = 0.0001 (1.03)
**Eprosartan (1997)**				
11 ^8 wks^	SiDBP	100.9/2.8 [87]	**101.0/8.0 [86]**	p = 0.00000112 (0.74)
13 ^13 wks^	SiDBP	102.6/4.4 [86]	**102.2/8.3 [78]**	p < 0.05 (0.43)
49 ^8 wks^	SiDBP	100.6/3.3 [72]	102.0/5.1 [70]	p = 0.121 (0.22)
			**101.5/6.2 [73]**	p = 0.0274 (0.36)
			100.7/5.9 [72]	p = 0.0934 (0.34)
			**100.6/7.6 [71]**	p = 0.0298 (0.53)
**Valsartan (1997)**				
10 ^4 wks^	SuDBP	100.7/4.6 [25]	101.6/6.7 [24]	p = 0.200 (0.36)
			**100.7/7.6 [22]**	p = 0.016 (0.47)
			**101.0/9.4 [24]**	p = 0.007 (0.78)
17 ^7 wks^	SuDBP	103.2/3.6 [57]	**103.7/8.6 [119]**	p = 0.001 (0.66)
			**104.0/6.9 [109]**	p = 0.046 (0.42)
31 ^8 wks^	SuDBP	100.7/2.3 [145]	**100.8/7.4 [148]**	P = 0.001 (0.66)
			**101.4/7.7 [147]**	P = 0.001 (0.73)
			**101.3/8.7 [150]**	p = 0.001 (0.78)
50 ^4 wks^	SuDBP	100.9/2.9 [183]	**100.8/7.8 [177]**	p = 0.0001 (0.61)
			**101.7/8.4 [187]**	p = 0.0001 (0.69)
51 ^8 wks^	SuDBP	101.8/5.3 [142]	**101.2/9.4 [136]**	p = 0.0001 (0.47)
**Telmisartan (1998)**				
502.202 ^4 wks^	SuDBP	104.0/1.5 [43]	**102.4/7.9 [40]**	p = 0.0059 (0.62)
			**101.7/8.7 [41]**	p = 0.0002 (0.84)
502.203 ^4 wks^	SuDBP	102.5/0.4 [46]	**101.5/8.6 [47]**	p = 0.0001 (0.84)
			**103.1/10.5 [44]**	p = 0.0001 (0.85)
502.204 ^8 wks^	SuDBP	100.3/4.3 [73]	**101.4/11.1 [75]**	p = 0.0001 (0.65)
			**100.3/11.8 [77]**	p = 0.0001 (0.65)
502.206 ^12 wks^	SuDBP	100.5/1.8 [74]	**100.4/9.3 [72]**	p = 0.0001 (0.66)
			**100.1/9.7 [71]**	p = 0.0001 (0.66)
502.207 ^8 wks^	SuDBP	100.4/2.7 [60]	**102.0/8.4 [59]**	p = 0.0001 (0.73)
502.208 ^12 wks^	SuDBP	101.4/4.5 [81]	**100.6/11.6 [73]**	p = 0.0001 (0.64)
**Candesartan (1998)**				
AM113 ^8 wks^	SiDBP	101.1/2.4 [63]	**100.1/7.6 [62]**	p = 0.0001 (0.72)
			**101.1/8.7 [60]**	p = 0.0001 (0.72)
			**100.1/7.9 [59]**	p = 0.0009 (0.61)
			**100.1/10.3 [57]**	p = 0.0001 (0.73)
EC009 ^4 wks^	SiDBP	--/3.4 [39]	--/6.5 [39]	p = n.s. (0.40)
			--/6.9 [39]	p = n.s. (0.46)
			--/8.7 [38]	p = n.s. (0.69)
			--/8.9 [38]	p = n.s. (0.69)
AHM0001 ^8 wks^	SiDBP	102.8/0.01 [85]	**101.7/8.4 [82]**	p = 0.001 (0.52)
			**102.5/9.4 [84]**	p = 0.001 (0.51)
EC047 ^12 wks^	SiDBP	102.9/2.0 [47]	**101.8/8.7 [50]**	p = 0.0001 (0.82)
			**102.2/7.8 [51]**	p = 0.0001 (0.81)
			**102.0/10.0 [51]**	p = 0.0001 (0.81)
			**101.9–10.3 [52]**	p = 0.0001 (0.81)
EC018 ^8 wks^	SiDBP	--/6.3 [44]	**--/10.1 [79]**	p = 0.0062 (0.55)
AM 116 ^8 wks^	SiDBP	99.9/3.3 [91]	**99.8/9.7 [93]**	p < 0.05 (0.67)
			**100.2/9.3 [90]**	p < 0.05 (0.65)
AHM0006 ^8 wks^	SiDBP	102.1/2.1 [83]	**102.1/10.0 [85]**	p = 0.001 (0.51)
			**103.0/9.1 [86]**	p = 0.001 (0.51)
EC011 ^12 wks^	SiDBP	103.6/5.3 [65]	103.5/8.4 [66]	p = 0.07 (0.32)
			**102.4/10.5 [68]**	p = 0.0024 (0.53)
			**103.3/10.0 [65]**	p = 0.0085 (0.47)
**Valsartan + HCT (1998)**				
301 ^8 wks^	SiDBP	101.4/4.1 [93]	**100.9/13.5 [96]**	p = 0.001 (0.49)
			**100.4/15.3 [91]**	p = 0.001 (0.49)
			**101.4/15.3 [94]**	p = 0.001 (0.49)
**Telmisartan + HCT (2000)**				
502.204 ^8 wks^	SuDBP	100.3/3.8 [73]	**101.1/14.9 [73]**	p < 0.05 (1.54)
			**100.6/14.4 [32]**	p < 0.05 (1.38)
			**100.5/12.7 [33]**	p < 0.05 (1.23)
			**101.1/18.0 [32]**	p < 0.05 (1.89)
**Eprosartan + HCT (2001)**				
061 ^8 wks^	SiDBP	101.0/5.4 [124]	**101.3/9.8 [128]**	p = 0.0001 (0.52)
			**99.8/12.2 [124]**	p = 0.0001 (0.81)
088 ^8 wks^	SiDBP	--/7.9 [156]	**--/10.7 [149]**	p < 0.05 (0.32)
016 ^4 wks^	SiDBP	100.4/4.9 [52]	**101.0/7.9 [53]**	p = 0.026 (0.44)
			**100.7/7.7 [51]**	p = 0.038 (0.41)
148 ^8 wks^	SiDBP	--/6.9 [119]	**--/11.9 [112]**	p < 0.05 (0.58)
**Candesartan + HCT (2001)**				
AHK0004 ^12 wks^	SiDBP	101.7/4.2 [93]	**101.3/12.8 [91]**	p = 0.001 (0.91)
EC408 ^12 wks^	SiDBP	99.9/7.1 [163]	**99.8/12.4 [165]**	p = 0.0001 (0.66)
AM153 ^8 wks^	SiDBP	100.9/3.7 [62]	**99.9/14.5 [63]**	p = 0.0001 (1.2)
AM124^12 wks^	SiDBP	100.2/5.4 [75]	**100.7/12.3 [154]**	p = 0.0001 (0.70)
EC403 ^8 wks^	SiDBP	102.0/4.0 [119]	**102.1/17.0 [39]**	p < 0.05 (1.23)
			**101.0/12.9 [43]**	p < 0.05 (0.88)
**Olmesartan (2002)**				
305 ^8 wks^	SiDBP	103.2/4.1 [88]	**103.6/11.5 [85]**	p = 0.001 (0.51)
			**103.6/11.9 [88]**	p = 0.001 (0.50)
10 ^12 wks^	SiDBP	104.6/10.2 [89]	**104.9/16.8 [166]**	p = 0.0001 (0.52)
9 ^12 wks^	SiDBP	103/9.2 [110]	**103/12.7 [112]**	p = 0.0001 (0.53)
			**103/14.4 [107]**	p = 0.0001 (0.54)
**Eplerenone (2002)**				
010 ^8wks^	SiDBP	101/1.1 [52]	**101/4.5 [54]**	p = 0.027 (0.42)
			**101/4.4 [48]**	p = 0.036 (0.40)
			**101/4.4 [53]**	p = 0.031 (0.41)
			**101/7.8 [53]**	p = 0.001 (0.83)
049 ^12 wks^	SiDBP	100/1.7 [87]	**101/4.6 [83]**	p = 0.011 (0.35)
			**100/6.3 [88]**	p = 0.0005 (0.54)
**Olmesartan + HCT (2003)**				
CS866-318 ^8 wks^	SiDBP	103/7.7 [42]	**104/15.4 [42]**	p = 0.0001 (0.90)
			**104/18.0 [42]**	p = 0.0001 (1.16)
			**104/18.9 [46]**	p = 0.0001 (1.47)
			**103/21.9 [39]**	p = 0.0001 (1.64)
**Amlodipine and Valsartan (2006)**				
A2201 ^8 wks^	SiDBP	99.4/6.8 [128]	**99.1/14.5 [128]**	p < 0.05 (--)
			**99.4/14.2 [127]**	p < 0.05 (--)
			**99.3/15.9 [127]**	p < 0.05 (--)
A2307 ^8 wks^	SiDBP	99.0/8.8 [209]	**99.2/17.6 [210]**	p = 0.0001 (0.38)
			**99.3/18.6 [219]**	p = 0.0001 (0.38)
**Amlodipine and Olmesartan (2007)**				
22100 ^8 wks^	SiDBP	102.4/3.1 [160]	**101.7/14.0 [160]**	p = 0.0001 (1.10)
			**100.9/15.5 [157]**	p = 0.0001 (1.30)
			**101.1/17.0 [158]**	p = 0.0001 (1.47)
			**102.3/19.0 [161]**	p = 0.0001 (1.62)
**Nebivolol hydrochloride (2007)**				
NEB302 ^12 wks^	SiDBP	100.3/2.9 [81]	**99.6/8.4 [165]**	p = 0.0001 (0.46)
			**99.5/9.2 [166]**	p = 0.0001 (0.57)
			**99.4/9.8 [166]**	p = 0.0001 (0.62)
			**99.3/11.2 [166]**	p = 0.0001 (0.75)
NEB305 ^12 wks^	SiDBP	98.7/4.6 [75]	**99.1/7.8 [244]**	p = 0.0015 (0.22)
			**98.9/8.5 [244]**	p = 0.0009 (0.26)
			**99.2/9.1 [244]**	p = 0.0002 (0.31)
**Aliskiren (2007)**				
1201 ^8 wks^	SiDBP	99.4/3.2 [115]	**99.5/7.7 [112]**	p = 0.0001 (0.54)
			**99.6/10.7 [113]**	p = 0.0001 (0.90)
2201 ^8 wks^	SiDBP	--/6.3 [130]	**--/9.3 [127]**	p = 0.0004 (0.35)
			**--/11.8 [130]**	p = 0.0001 (0.64)
2203 ^8 wks^	SiDBP	--/8.6 [176]	--/10.3 [177]	p = 0.0506 (0.21)
			**--/-12.3 [175]**	p = 0.0001 (0.45)
2204 ^8 wks^	SiDBP	--/6.9 [192]	**--/8.9 [183]**	p = 0.0152 (0.25)
			**--/10.3 [180]**	p = 0.0001 (0.42)
2308 ^8 wks^	SiDBP	--/4.9 [163]	**--/10.3 [167]**	p = 0.0001 (0.66)
			**--/11.1 [166]**	p = 0.0001 (0.75)
2327 ^8 wks^	SiDBP	--/4.1 [455]	**--/9.0 [430]**	p = 0.0001 (0.56)
**Aliskiren HCT (2007)**				
2204	SiDBP	--/6.9 [192]	**--/11.9 [184]**	p = 0.0001 (0.62)
			**--/12.7 [187]**	p = 0.0001 (0.72)
			**--/13.7 [180]**	p = 0.0001 (0.84)
			**--/14.3 [173]**	p = 0.0001 (0.92)
**Aliskiren and Valsartan (2009)**				
2327 ^8 wks^	SiDBP	--/4.1 [455]	**--/12.2 [438]**	p = 0.0001 (0.93)
**Amlodipine and Telmisartan (2009)**				
1235.1^8 wks^	SiDBP	102.5/5.9 [46]	**102.3/18.0 [44]**	p < 0.05 (1.39)
			**102.7/15.7 [45]**	p < 0.05 (1.20)
			**100.8/18.7 [40]**	p < 0.05 (1.52)
			**101.1/16.2 [47]**	p < 0.05 (1.16)
			**101.6/16.0 [141]**	p < 0.05 (1.25)
			**101.6/19.6 [123]**	p < 0.05 (1.64)
			**101.4/15.3 [46]**	p < 0.05 (1.10)
			**101.8/17.8 [143]**	p < 0.05 (1.36)
			**101.3/19.6 [136]**	p < 0.05 (1.64)
**Aliskiren and Amlodipine (2010)**				
2305 ^8 wks^	SiDBP	99.6/5.4 [198]	**99.9/14.0 [179]**	p = 0.001 (0.98)
			**99.4/16.2 [179]**	p = 0.001 (1.23)
			**99.6/15.0 [175]**	p = 0.001 (1.10)
			**99.5/16.5 [183]**	p = 0.001 (1.27)
**Azilsartan (2011)**				
01-05-TL-491-008 ^6 wks^	ambDBP	87.2/0.69 [142]	**88.0/8.4 [281]**	p = 0.001 (1.00)
			**87.7/8.6 [284]**	p = 0.001 (1.03)
01-06-TL-491-019 ^6 wks^	ambDBP	88.7/0.07 [154]	**87.9/8.7 [280]**	p = 0.001 (1.10)
			**88.6/9.4 [283]**	p = 0.001 (1.17)
01-05-TL-491-005 ^8 wks^	SiDBP	100.1/7.9 [61]	**99.7/13.6 [62]**	p = 0.001 (0.65)
			**100.3/11.6 [64]**	p = 0.018 (0.42)
**Perindopril and Amlodipine (2015)**				
005 ^8 wks^	SuDBP	100.5/9.3 [248]	**100.7/13.6 [246]**	p = 0.001 (0.47)
**Nebivolol and Valsartan (2016)**				
MD-01 ^8 wks^	SiDBP	99.8/7.1 [277]	**99.6/15.0 [549]**	p = 0.0001 (0.82)
			**99.6/15.1 [548]**	p = 0.0001 (0.84)
			**99.9/15.7 [550]**	p = 0.0001 (0.90)

^a^ bold treatment arms indicate success (p< 0.05).

Table 1 presents the essential characteristics and raw data of 142 antihypertensive treatment arms organized by year of approval. Of the 142 treatments arms for antihypertensive medications, 25 p-value thresholds were given instead of exact p-value and therefore effect size estimates were calculated using the traditional formula for Hedges’ G using standard deviation. Additionally, three treatment arms did not have enough data (either an exact p-value or standard deviations) to calculate treatment effect sizes (see Table 1).

As shown in Table 1, 100% (63/63) of the trials used diastolic blood pressure as the primary outcome measure. In-office seated blood pressure measurement was used in the majority of the trials (69.8%; 44/63) while in-office measurement with the patient supine was used in 27.0% (17/63) of the trials. A very small percentage (3.2%; 2/63) used out-of-office, ambulatory blood pressure monitoring as the trial’s primary outcome measure.

### Placebo and antihypertensive response over time

**Fig 1:**
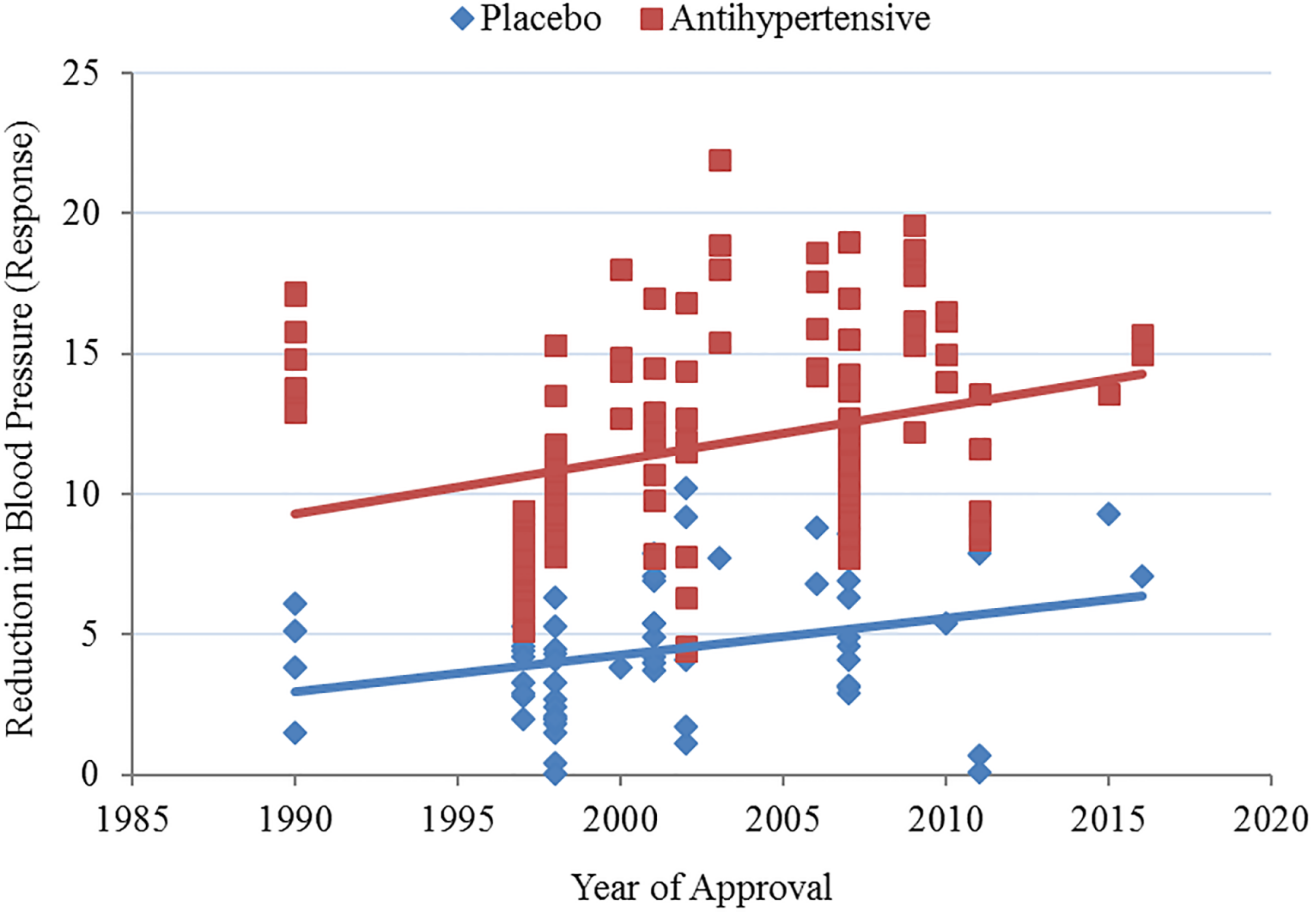
Antihypertensive and placebo response (blood pressure reduction) plotted with year of approval.

As can be seen in Fig 1, placebo response appears to be increasing over time. A simple meta-regression was modeled to predict placebo response based on year of approval and significance was found (p = 0.013) with an *R*^2^ of 0.093. Placebo response increased by 0.131 for each year following 1990.

Antihypertensive response similarly increased with year of approval (see Fig 1) and significance for the meta-regression model was found (p<0.001), with an *R*^2^ of 0.086. Antihypertensive response increased by 0.193 for each year following 1999.

### Efficacy outcomes (effect size, drug-placebo difference, and success rate) over time

**Fig 2:**
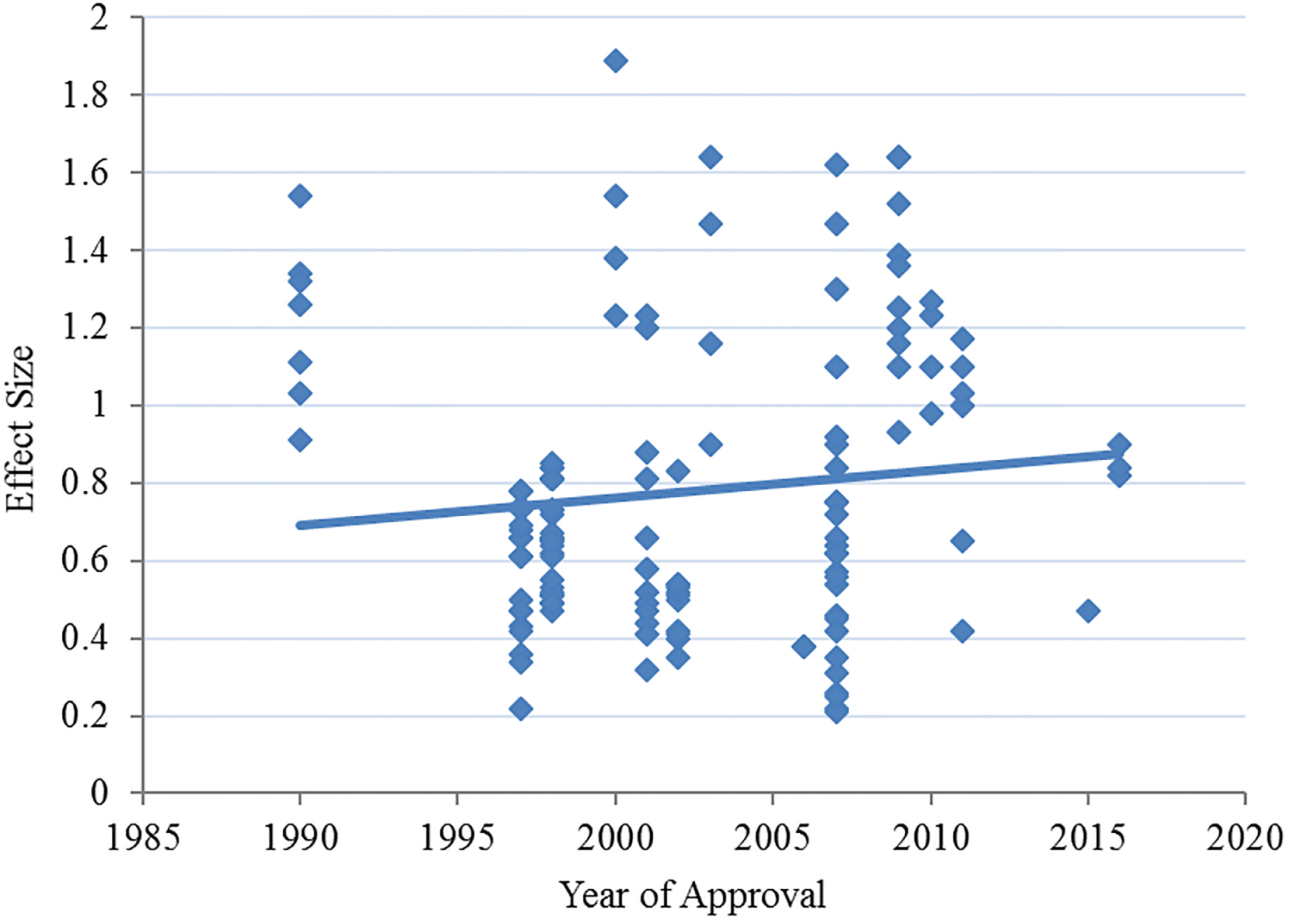
Scatterplot of effect sizes for 139 treatment arms from 62 clinical trials of investigational medication approval programs plotted with year of approval.

The meta-regression analysis revealed that the apparent increase in effect size over time was not significant (see Fig 2) (*R*^2^ = 0.017, p = 0.119). A lack of significant change over time was also evident in the regression analysis of antihypertensive-placebo response differences (*R*^2^ = 0.013, p = 0.176). Overall, antihypertensives maintain superiority over placebo by about 7.2 (±3.1) diastolic blood pressure points with a mean effect size of 0.78 (±0.37). The rate of statistical superiority of drug over placebo (success rate, as determined by the statistical analysis of the FDA reviewer) for the treatment arms we analyzed was 94.4% (134/142) and did not change over time.

### Placebo response and antihypertensive efficacy outcomes

Placebo response had no relationship to effect size (β = -0.002, *R*^2^ = 0.0002, p = 0.877), antihypertensive-placebo differences (β = -0.035, *R*^2^ = 0.001, p = 0.752), or p-values (β = 0.0001, *R*^2^ = 0.0003, p = 0.963), showing that despite the increase in magnitude of placebo response, there has been no impact on clinical trial efficacy outcomes.

However, the rise in placebo response was significantly related to the rise in antihypertensive response (β = 0.965, *R*^2^ = 0.347, p<0.0001) indicating that as the reduction in blood pressure points went up in the placebo treatment group, the reduction in blood pressure with antihypertensives increased by nearly the same amount.

### Relationship of trial characteristics to placebo/drug response and efficacy outcomes

The *R*^2^ of the model predicting placebo response as a factor of the duration, number of treatment arms, placebo baseline blood pressure, and treatment arm sample size was 0.30 (p = 0.0003). Out of these variables, higher placebo baseline blood pressure (β = 0.364, p = 0.0007) and treatment arm sample size (β = 0.008, p = 0.0019) significantly predicted higher placebo response. When examined independently, only sample size remained statistically significant (β = 0.004, *R*^2^ = 0.072, p = 0.0312).

The model predicting drug response as a factor of the same variables was also significant, with an *R*^2^ = 0.482 (p<0.0001). Higher drug baseline blood pressure (β = 0.325, p = 0.0012), greater number of treatment arms (β = 0.598, p<0.0001), and a larger sample size (β = 0.004, p = 0.0165) significantly predicted higher response to antihypertensive treatment. When examined independently, only drug baseline blood pressure (β = 0.284, *R*^2^ = 0.039, p = 0.027) and number of treatment arms (β = 0.52, *R*^2^ = 0.351, p<0.0001) remained statistically significant.

For the model predicting the efficacy outcome of treatment arm standardized effect sizes (Hedges’ G), we entered in the duration of the trial, number of treatment arms, treatment arm sample size, and the baseline blood pressure for each treatment arm overall (weighted average of placebo and drug treatment group baselines). The overall model had an *R*^2^ of 0.421 (p<0.0001). Duration (β = -0.038, p = 0.0003), number of treatment arms (β = 0.059, p<0.0001), and the treatment arm overall baseline blood pressure (β = -0.021, p = 0.044) were significantly related to the treatment effect size. When examined individually, only duration (β = -0.041, *R*^2^ = 0.07, p = 0.0018) and number of treatment arms (β = 0.042, *R*^2^ = 0.213, p<0.0001) remained significant, with shorter duration and higher number of treatment arms predicting higher effect size.

### Trial characteristics over time

**Fig 3:**
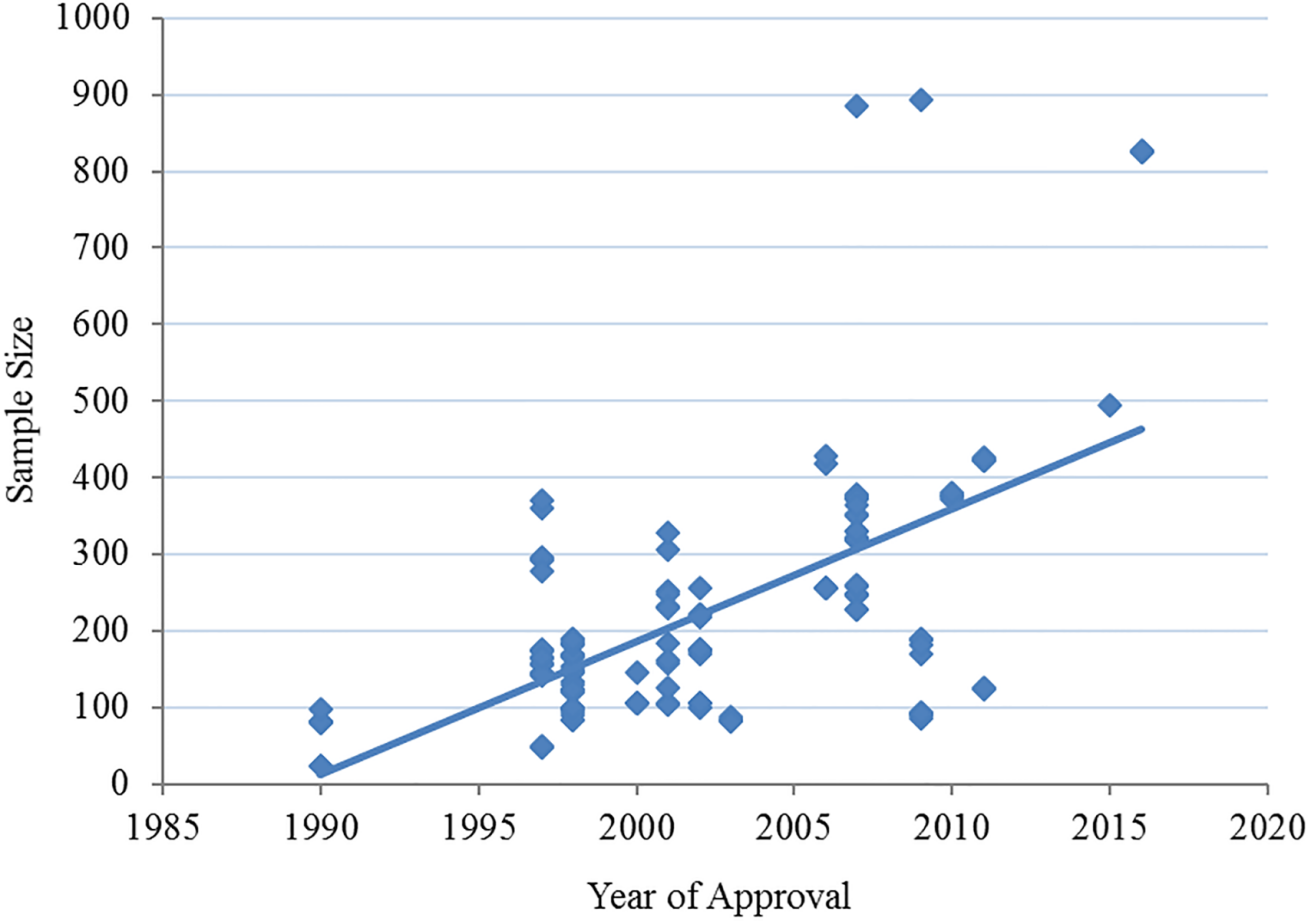
Scatterplot of 142 antihypertension treatment arm sample sizes plotted with year of approval.

Sample size has increased significantly over time (p< 0.0001) with an *R*^2^ = 0.387 (see Fig 3). Each year following 1990, the sample size increased by about 17.3 patients per treatment arm comparison, with a range of 23–370 N before 2005 and 86–893 N after 2005.

The number of treatment arms also increased significantly over time (β = 0.247, *R*^2^ = 0.176, p = 0.0003) while the treatment arm baseline blood pressure decreased significantly (β = -0.213, *R*^2^ = 0.21, p<0.0001). Duration did not change significantly over time (*R*^2^ = 0.047, p = 0.08).

## Discussion

This study evaluated clinical trial data from FDA reviews of antihypertensive medications with the aim of testing if the pattern of a rising placebo response and stable efficacy outcomes seen over time in trials of psychiatric [4,5] and medical conditions [6,7] could be seen in clinical trials for hypertension. As hypothesized, antihypertensive clinical trial data showed a similar pattern to the psychiatric and medical trials previously analyzed, wherein placebo response increased significantly over time and outcome measures of effect size, drug-placebo differences, and success rate remained the same, likely due to the parallel and significant increase in active treatment response. As confirmed by the lack of relationship between the magnitude of placebo response and trial efficacy outcomes, it appears that growth in placebo response over time did not have any impact on antihypertensive clinical trial efficacy outcomes.

It is interesting that clinical trials evaluating medications for non-psychiatric conditions using physiologically-measured endpoints, as the hypertension trials do, exhibit the same dramatic increase in placebo response over time as the other conditions, nearly doubling over 25 years of antihypertensive trial history (see Fig 1). Although with retrospective data it is not possible to determine a causal explanation, one possible explanation for the rising placebo response is that given the historical shift towards direct-to-consumer marketing of prescription medications, patients may have higher expectancy for medication effects. Although conceptually sound and based on observed evidence [22], this theory has not been tested prospectively.

Furthermore, it is likely that the nearly 50% increase in drug response (see Fig 1) is due to the additive nature of placebo response, which inherently contributes to the measurement of the overall drug effect. While the overall efficacy of the agents appears stable, as seen by the constant distance between the drug and placebo response and stable effect sizes (see Figs 1 and 2), the proportion of the drug response that represents nonspecific placebo effects has likely increased over time parallel to the placebo treatment arms. This is supported by our finding of a significant correlation (*R*^2^ = 0.347, p<0.0001) between the magnitude of blood pressure reduction with placebo and with antihypertensives. While the additive relationship between placebo and drug response in the measure of antihypertensive response is assumed, it is important to test this assumption in light of the attention and concern given to the rising placebo response. What these data show is that there has not been a ceiling effect on the response to antihypertensive agents and that the growth in drug response likely reflects a growth in placebo response. Considered collectively, these findings suggest that efforts to reduce or control the response to placebo in antihypertensive clinical trials may not be necessary for successful efficacy outcomes.

In our exploratory analysis, we evaluated the potential role of the duration, treatment arm sample size, baseline blood pressure, and number of treatment arms on efficacy outcomes, placebo, and drug response. The results were mixed and the trial and/or patient characteristics that surfaced were not reliable. Higher baseline blood pressure did appear to have some potential relationship to higher placebo and drug response. This may potentially be related to increased effects from regression to the mean, with initially more severe cases of hypertension returning to average over the course of the trial. Greater number of treatment arms appeared to have a relationship with higher drug response and effect size and the number of treatment arms also increased over time. This finding is disjointed from previous analyses that have indicated that greater number of treatment arms (as a measure of the likelihood of a patient receiving placebo, or in other words, patient expectations) may increase the placebo response [23,24]. Shorter trial duration appeared to predict higher effect sizes and the average trial duration did not change significantly over the time period examined. Higher treatment arm sample size appeared to predict higher placebo response, although the size of the effect (β = 0.004, *R*^2^ = 0.072, p = 0.0312) was quite small.

While these findings may inform future trial design, it is important to note that these data are from a multivariate meta-regression based on retrospective analysis which can be subject to spurious findings. Additionally, all of the factors when examined together accounted for less than half of the variance in effect size and placebo/drug responses, suggesting the influence of variables that we were not able to quantify with these data. Finally, these findings do not offer a coherent explanation for the rise in placebo response and stability in efficacy outcome measures: for example, baseline blood pressure has decreased significantly over time, which should have predicted a decreasing placebo and drug response over time based off of the meta-regression findings.

What is clear from these data is that these trials are well over-powered to demonstrate the average effect size (0.78 ±0.37: ~50N required between placebo and active treatment for a statistical power of 85%). The mean trial arm sample size (~350N) exceeds the required number of patients by seven times and in some trials, up to 15 times over. The trend of overpowering is continuing as sample size has increased significantly over time (*R*^2^ = 0.387, p<0.001, see Fig 3), while effect sizes (see Fig 2) and antihypertensive-placebo differences have not changed significantly—indicating that while there has been no demonstration of better or worse efficacy among antihypertensive trials, there has been significantly more patient exposure to the research paradigm.

One possible explanation for this observation is that more recent trials may be designed to serve dually as both efficacy and safety evaluations, requiring greater patient exposure. Another potential explanation is that regulatory and publication agencies may still view trials with ~50N per treatment arm as small, even despite the reliably large effect size, and may be reluctant to accept the findings. Overpowering may protect against this bias as well as ensure that even if the treatment effect found is smaller than expected, that the treatment arm will still be successful. While such overly adequate powering may help explain the fact that trial outcomes have remained unaffected while placebo response has increased, excessive exposure and use of resources should also be considered.

Additionally, while techniques like 24-hr ambulatory blood pressure monitoring have been shown to increase reproducibility [13–15] and yield lower estimations of placebo response [16–19], their adoption as primary efficacy endpoints in FDA clinical trials for hypertension has not been widespread. Only two out of 63 trials (3.2%) used such a technique as a primary outcome measure. Although not prospectively tested in clinical trials, the lower variance in placebo and drug response associated with the use of ambulatory monitoring may require fewer patients to demonstrate equivalent treatment efficacy.

The limitations of this study include the fact that it is retrospective analysis and patient-level data are not available in these summary reviews. Additionally, our statistical analysis of trial and patient variables was limited because there was low reporting for patient characteristics and little representation of primary outcome measures other than diastolic, in-office measurement. Finally, these data represent the statistical analyses of FDA reviewers examining efficacy trials for investigational medications that eventually received approval. Therefore, these data do not represent the full spectrum of trials using investigational antihypertensive agents. While selection bias does not occur in the same way that it does in published studies (in that FDA reviewers evaluate all trials conducted for efficacy regardless of positive or negative outcome), there are likely biases stemming from regulatory processes (including the type of statistical analysis and only reporting on trials deemed to be of sufficient quality of conduct and design).

This study provides evidence that the magnitude of placebo response is rising in FDA clinical trials for hypertension, similar to what has been observed in trials for several other medical and psychiatric conditions. Like antidepressants, ADHD medications, antiepileptics, and antihyperglycemics, this rise in placebo response has not negatively impacted efficacy outcomes including standardized treatment effect size, raw drug-placebo difference in blood pressure reduction, and success rate. As expected in adequately powered trials, the drug response has also increased and the magnitude of placebo response shows no relationship to outcomes of effect size, drug-placebo difference, or p-values, suggesting that attempts to control the response to placebo may not be necessary. Among the trial design and patient variables that we could quantify, there was not a clear explanation for the phenomenon of the rise in placebo response and stability in efficacy outcomes over time. These data suggest that the phenomenon of increasing placebo response and stable efficacy outcomes may be a general trend, occurring across trials for various psychiatric and medical conditions with physiological and non-physiological endpoints.
